# Impact of the *Mycobaterium africanum* West Africa 2 Lineage on TB Diagnostics in West Africa: Decreased Sensitivity of Rapid Identification Tests in The Gambia

**DOI:** 10.1371/journal.pntd.0004801

**Published:** 2016-07-07

**Authors:** Boatema Ofori-Anyinam, Fatoumatta Kanuteh, Schadrac C. Agbla, Ifedayo Adetifa, Catherine Okoi, Gregory Dolganov, Gary Schoolnik, Ousman Secka, Martin Antonio, Bouke C. de Jong, Florian Gehre

**Affiliations:** 1 Mycobacteriology Unit, Institute of Tropical Medicine (ITM), Antwerp, Belgium; 2 Vaccines and Immunity Theme, Medical Research Council (MRC) Unit, Serrekunda, The Gambia; 3 Disease Control and Elimination Theme, Medical Research Council (MRC) Unit, Serrekunda, The Gambia; 4 Department of Infectious Diseases Epidemiology, London School of Hygiene & Tropical Medicine, London, United Kingdom; 5 Department of Microbiology and Immunology, Stanford University School of Medicine, Stanford University, Stanford, California, United States of America; 6 Division of Microbiology & Immunity, Warwick Medical School, Coventry, United Kingdom; 7 Faculty of Infectious and Tropical Diseases, London School of Hygiene & Tropical Medicine, London, United Kingdom; 8 Division of Infectious Diseases, New York University, New York, New York, United States of America; Fondation Raoul Follereau, FRANCE

## Abstract

**Background:**

MPT64 rapid speciation tests are increasingly being used in diagnosis of tuberculosis (TB). *Mycobacterium africanum* West Africa 2 (*Maf 2*) remains an important cause of TB in West Africa and causes one third of disease in The Gambia. Since the introduction of MPT64 antigen tests, a higher than expected rate of suspected non-tuberculous mycobacteria (NTM) was seen among AFB smear positive TB suspects, which led us to prospectively assess sensitivity of the MPT64 antigen test in our setting.

**Methodology/Principal Findings:**

We compared the abundance of mRNA encoded by the *mpt64* gene in sputa of patients with untreated pulmonary TB caused by *Maf* 2 and *Mycobacterium tuberculosis (Mtb)*. Subsequently, prospectively collected sputum samples from presumptive TB patients were inoculated in the BACTEC MGIT 960 System. One hundred and seventy-three acid fast bacilli (AFB)-positive and blood agar negative MGIT cultures were included in the study. Cultures were tested on the day of MGIT positivity with the BD MGIT TBc Identification Test. A random set of positives and all negatives were additionally tested with the SD Bioline Ag MPT64 Rapid. MPT64 negative cultures were further incubated at 37°C and retested until positive. Bacteria were spoligotyped and assigned to different lineages. *Maf* 2 isolates were 2.52-fold less likely to produce a positive test result and sensitivity ranged from 78.4% to 84.3% at the beginning and end of the recommended 10 day testing window, respectively. There was no significant difference between the tests. We further showed that the decreased rapid test sensitivity was attributable to variations in mycobacterial growth behavior and the smear grades of the patient.

**Conclusions/Significance:**

In areas where *Maf 2* is endemic MPT64 tests should be cautiously used and MPT64 negative results confirmed by a second technique, such as nucleic acid amplification tests, to avoid their misclassification as NTMs.

## Author Summary

Diagnostics for rapid confirmation of positive liquid cultures presumptive of *Mycobacterium tuberculosis* bacteria, based on the detection of the MPT64 antigen, are being used in many TB diagnostic laboratories worldwide. Of note, diagnostic performance of these tests in West Africa, where TB is uniquely caused by the geographically restricted *Mycobacterium africanum* (*Maf 1* and *2*) and *Mycobacterium tuberculosis* lineages, has not been properly assessed. Although *M*. *tuberculosis* and *M*. *africanum* are genetically related, they differ in various aspects. Amongst several differences, *Maf 2* grows significantly slower than *Mtb* bacteria. Because secretion of the MTP64 protein is dependent on the bacterial growth rate, we found that the MPT64 rapid test performance for detecting *Maf 2* was lower in our setting in The Gambia. These findings might be relevant for other West African *Maf 2* endemic countries where this rapid test is commonly used, as *Maf 2* infected patients might have been missed in the past. Our finding emphasizes the need to thoroughly consider the presence of bacterial variants specific to certain regions during product development and implementation of novel diagnostic tests.

## Introduction

Tuberculosis remains a significant public health problem in Africa. Key to interrupting transmission, which is an essential step to reduce the incidence of TB, is timely identification and treatment of diseased individuals. This objective has fueled research into new generations of diagnostics and drugs. MPT64 is a 24kD secreted protein that has been explored in diagnostics and vaccine design due to several properties: it has been associated with virulence, is highly immunogenic and is produced solely by members of the *Mycobacterium tuberculosis* complex [1,2,3]. MPT64 is the target of three widely used rapid speciation lateral flow assays for the identification of the MTBc in culture; BD MGIT TBc Identification Test (BD TBc ID) (Becton Dickinson Diagnostics, Becton, Dickinson and Company, Sparks, Maryland, USA), SD Bioline Ag MPT64 Rapid (SD Bioline) (Standard diagnostics, Inc., Yongin-si, Gyeonggi-do, Republic of Korea), and Capilia TB-Neo (TAUNS Laboratories, Inc., Numazu, Shizuoka, Japan). Despite the advantage of a lateral flow assay, there have been reports of the failure of MPT64 tests to detect MTBc isolates, resulting in erroneous reporting of Non-tuberculous mycobacteria (NTM) isolation [2,3,4,5]. Inability to identify the MTBc will lead to delays in initiating appropriate treatment with dire consequences for the patient and their communities given the continued risk of TB transmission.

The global phylogeographical distribution of the MTBc suggests that strain diversity is greatest in West Africa, with a representation of all major MTBc lineages [6]. Differences between these MTBc lineages might affect the performance of diagnostics and vaccines [7]. Thus, West Africa is ideal for providing a global snapshot of the performance of TB diagnostics and vaccines. Interestingly, few studies assessing commercially available MPT64 MTBc rapid speciation tests were undertaken within African populations. They were mostly done in Asia, America and Europe where strain diversity is significantly lower [8].

Lineage 6, *Mycobacterium africanum* (*Maf*) West African 2, is geographically restricted to West Africa, causing up to one third of clinically reported TB. We and others have previously described inherent genotypic and phenotypic differences between *Mycobacterium tuberculosis* sensu stricto (*Mtb*) and *Maf 2* strains *in vitro* and within the host [9,10,11,12]. Since we previously observed differences in various virulence factors between *Maf 2* and *Mtb* and MPT64 is a described virulence factor, we hypothesized that the sensitivity of MPT64 rapid tests for MTBc identification could be different for *Maf 2* relative to *Mtb* strains.

In The Gambia, where *Maf* 2 is commonly isolated, we compared the abundance of mRNA encoded by the *mpt64* gene in *Maf* 2 strains versus *Mtb* strains in the sputa of untreated TB patients. The *mpt64* (Rv1980c) mRNA transcript was significantly less abundant in the sputa of TB patients infected with *Maf* 2 compared with *Mtb*. Therefore we concluded that *Maf 2* might either produce the MPT64 protein at a slower rate and possibly below the limit of detection of the rapid tests. We confirmed this hypothesis and found a reduced *Maf 2* sensitivity of rapid tests. Further, we compared the time to detection of the MPT64 antigen by the BD TBc ID and SD Bioline rapid tests between clinical isolates of *Mtb* and *Maf 2*. We report lineage dependent and time specific differences in conversion to MPT64 test positivity. Our findings have direct implications on the performance of these and other MPT64 based tools in West Africa.

## Materials and Methods

### Ethics Statement

This study was nested within an intervention trial of Enhanced Case Finding in The Gambia (Clinicaltrials.gov NCT01660646). The parent study, including bacterial sub-studies, received ethical approval from the Joint Gambia Government/MRC Ethics Committee and the Institute of Tropical Medicine (ITM), Antwerp Institutional Review Board. Written informed consent was obtained from all participants who were assigned unique identifiers for purposes of anonymity and confidentiality.

### Microscopy and Decontamination of Sputum

Sputum samples were prospectively collected from individuals with suspected TB between April and October 2014 and were all initially screened for the presence of AFB by Auramine microscopy. Fresh samples were decontaminated by the NALC-NaOH method as described previously [13]. The purity of all decontaminated samples was subsequently checked on blood agar for 48 hours at 37°C and screened for AFB by Ziehl-Neelsen [14] staining, during which time the decontaminated sputa were stored at -20°C, prior to inoculation.

### *Mtb* Gene Expression Analysis in Sputum

Sputa from 11 adult patients with smear positive TB that had not started therapy were collected and stored in Guanidine Isothiocyanate. Later 5 *Maf 2* and 6 *Mtb* sputum specimens were re-suspended in Trizol for total RNA isolation. Gene expression of *mpt64* gene was performed using Multiplex qPCR with previously published TaqMan primer-probe sets as described previously [15,16]. Multiplex RT-PCR data were normalized and analysed according to a previously published method. For a detailed description please refer to Garcia *et al*. [17].

### Bacterial Culture

Confirmed blood agar negative and AFB positive samples were cultured within the BACTEC MGIT 960 System (MGIT 960; Becton Dickinson Microbiology Systems, Sparks, Maryland, USA) at 37°C according to the manufactures’ instructions. Instrument positive vials were removed from the machine and again subjected to purity check using blood agar and ZN microscopy to confirm the presence of AFB. Cultures with growth on blood agar and/or AFB negative were excluded from the study.

### MPT64 Antigen Test

Blood agar negative and AFB positive MGIT cultures were tested on the day of MGIT positivity (day 0, T_0_) with the BD TBc ID rapid speciation lateral flow assay, following the manufacturers’ instructions. The BD TBc ID manual specifies that tests should be performed on AFB-positive MGIT tubes only if AFB-positive organisms predominate on a smear and cautions on the possibility of false results due to the presence of non-AFB organisms in cultures. In order to test whether the lower sensitivity of the MPT64 assay was specific to one manufacturer, we additionally tested all BD TBc ID negative samples at T_0_, including a random number of positive samples, with the SD Bioline kit at the same time points. Tests were always independently evaluated by at least two blinded readers; in case of discordance between the readers a third blinded reader was consulted. Faint bands were considered as positive results. Results were recorded after 15 minutes and negative cultures at T_0_ were retested at 3, 10, 15 and 90 days with both the BD TBc ID and SD Bioline rapid speciation kits.

### Spoligotyping

Aliquots of the MGIT cultures were taken at T_0_ and heat killed. These lysates were genotyped by spoligotyping as previously described [18]. Patient isolates were assigned to specific TB lineages using the TB-lineage tool [19] within the TB-Insight public database.

### Statistical Analysis

We plotted the survival curves for *Maf* 2 and *Mtb* conversion to rapid test positivity at 1, 3, 10, 15 and 90 days and used the generalized Log-rank test for interval-censored failure time [20] to compare the two survivor functions. We estimated the effect of MTBc lineage (*Maf 2* and *Mtb*) on the time-to-conversion to rapid test positivity using a Weibull regression model for interval-censored failure time [20] after controlling for testing time interval, age, gender, smear grade, duration spent in the MGIT, mycobacterial growth units, and whether patients had already initiated therapy. The BD TBc ID manual indicated that tests could be performed within 10 days after MGIT tube positivity, while SD Bioline did not specify any time interval for the performance of tests. Therefore, for comparative evaluation we stratified both analyses by a 0–10 days testing window and 10–90 days follow-up. A paired interval-censored analysis was used to compare BD TBc ID and SD Bioline performance. All analyses were performed using STATA 13.1 (Stata Corp., College Station, Texas).

## Results

### Lower Expression of the *mpt64* Transcript in the Sputa of *M*. *africanum* Compared to *M*. *tuberculosis* TB Patients

On comparing the abundance of the *mtp64* mRNA transcript in sputum samples from 6 *Mtb* and 5 *Maf 2* infected patients, who had not received any TB treatment, the *mpt64* transcript was significantly less abundant in the sputa of *Maf 2*-infected patients, compared to sputa of *Mtb*-infected patients (fold change = 2.52, p = 0.006). To validate our expression data, we measured the abundance of mRNA transcripts of the gene (Rv2355) responsible for the transposition of IS6110 elements in *Maf 2* samples since *Maf 2* is known to have lower copy numbers of IS6110 elements than *Mtb* [21]. Interestingly, we found a significantly reduced abundance of mRNA transcripts of this transposase in *Maf 2* samples. *In vitro* under expression of the polyketide synthesis loci responsible for production of sulfolipids in *Maf 2* has been reported [22], which were also significantly downregulated in *Maf 2* in our *ex vivo* data. For a detailed overview of mRNA expression for each individual patient please see S3 Table.

### Culture of Bacteria

**Fig 1:**
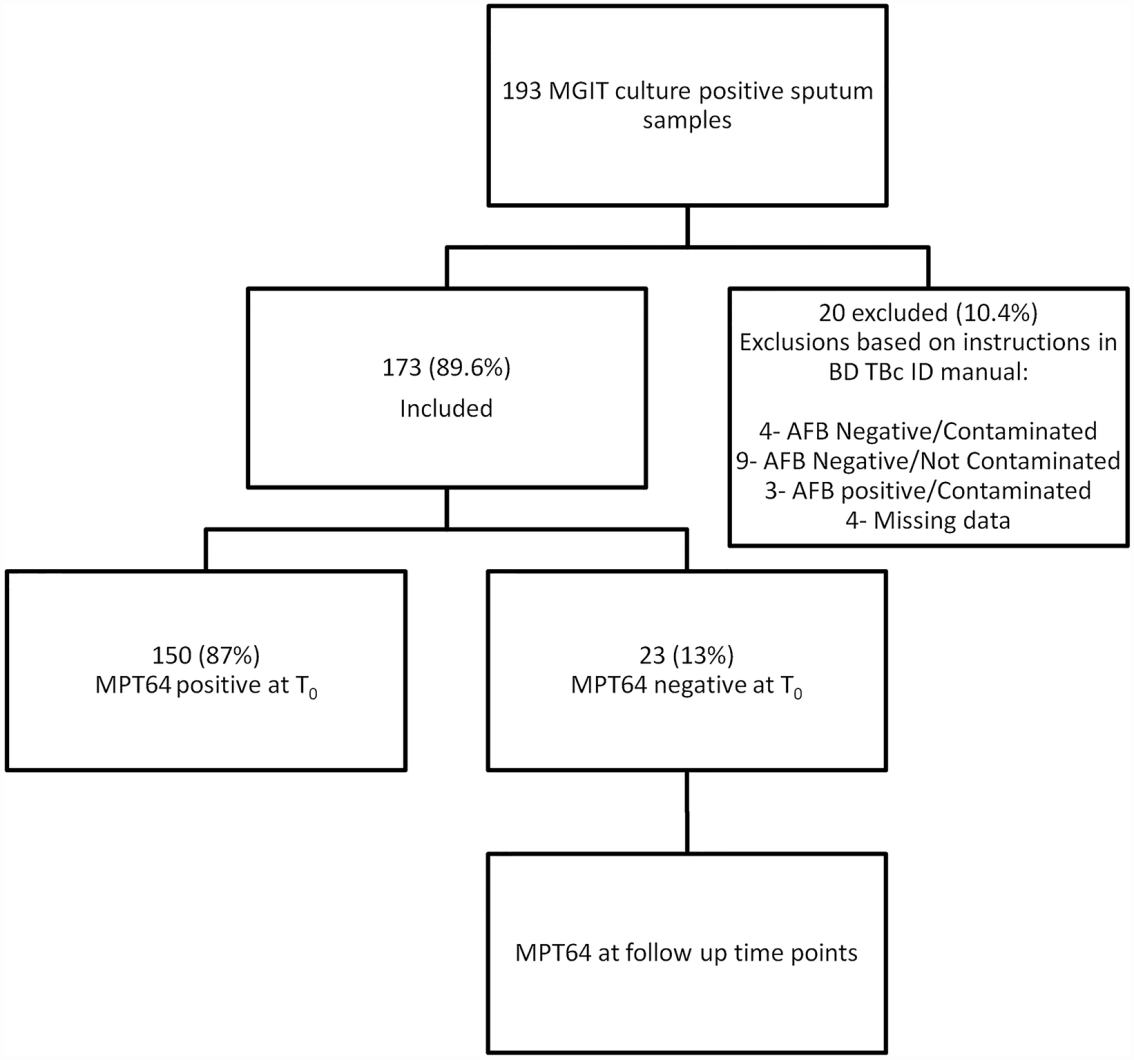
Flowchart describing sample collection and processing.

Altogether, 193 sputum samples from 193 presumptive TB patients were cultured in the MGIT 960 System. For a summary of patients’ characteristics see Table 1. Of the 193 cultures, 20 (10.4%) were excluded from the study due to missing patient data (n = 4; (2.1%)), being AFB negative and contaminated with other microorganisms on blood agar (n = 4; (2.1%)), AFB negative without contamination (n = 9; (4.7%)), or AFB positive and contaminated (n = 3; (1.5%)) (see Fig 1).

**Table 1:** Clinical and demographic characteristics of study participants.

	*Maf 2*	*Mtb*
	(n = 51)	(n = 122)
Age, median (range)	30 (17–67)	30 (15–90)
Gender		
Male, n (%)	46 (90.2)	79 (64.8)
Female, n (%)	5 (9.8)	43 (35.2)
Smear grade, n (%)		
Scanty	4 (7.8)	15 (12.3)
+	14 (27.5)	28 (23.0)
++	11 (21.6)	37 (30.3)
+++	22 (43.1)	42 (34.4)
Therapy, n (%)		
Yes	24 (47.1)	60 (49.2)
No	10 (19.6)	18 (14.8)
Unknown	17 (33.3)	44 (36.1)

A hundred and seventy-three positive cultures (89.6%), from 168 AFB smear positive and 5 AFB smear negative sputa, were blood agar negative, AFB positive by ZN staining and were tested with the BD TBc ID kit (Fig 1). One hundred and fifty samples tested positive with the BD TBc ID speciation kit on T_0_, with 23 testing negative at this time point, representing 13.2% of cultures tested.

### Lower Sensitivity of MPT64 Tests for *M*. *africanum* than *M*. *tuberculosis*

All 173 samples were spoligotyped and assigned to a lineage within the MTBc. Samples genotyped belonged to lineages 1 (Indo-oceanic), 2 (East-Asian/Beijing), 4 (Euro-American) or 6 (*Maf 2*).

The conversion time of the BD TBc ID assay did not differ among the three *Mtb* lineages- East-Asian, Euro-American and Indo-Oceanic (East-Asian and Euro-American (p = 0.55), East-Asian and Indo-Oceanic (p = 0.89) and Euro-American and Indo-Oceanic (p = 0.63) (S1 Table). Therefore, we combined the three lineages into one group, *Mtb*.

**Fig 2:**
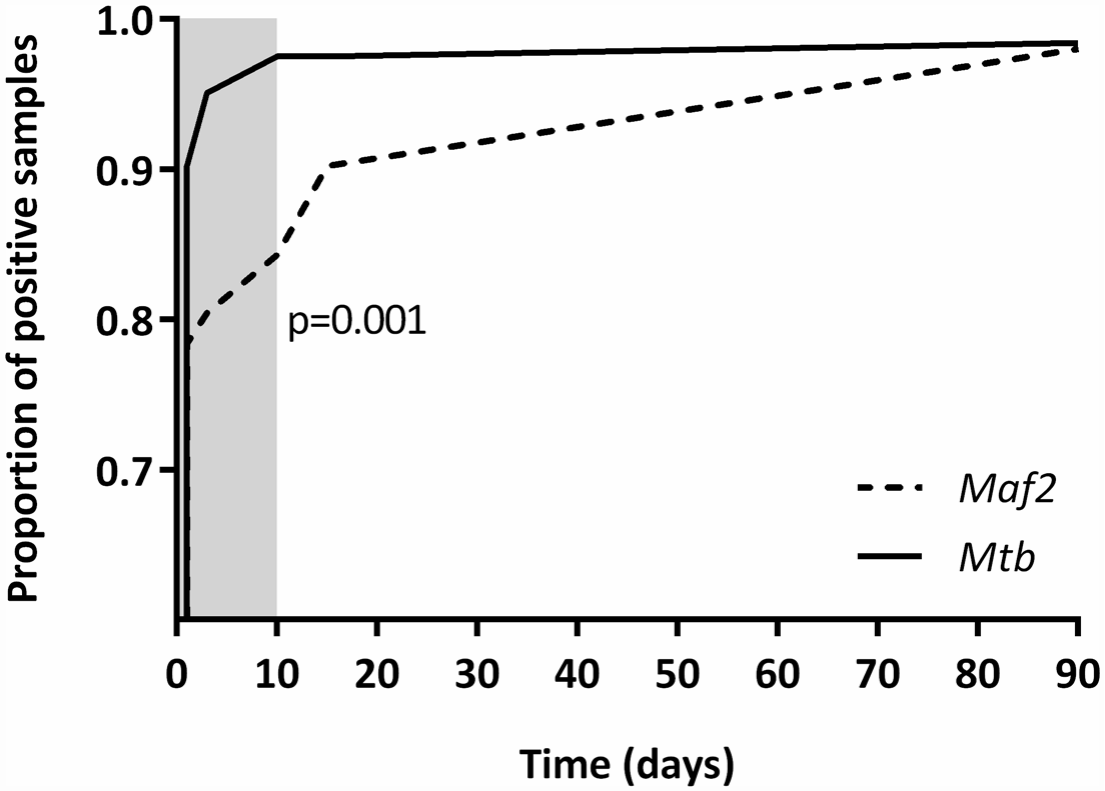
Kaplan-Meier survival plot showing the difference in conversion to rapid test positivity for *Mtb* strains and *Maf 2*. The Log-rank test was used to compare the survivor functions of *Mtb* and *Maf 2*. The gray shaded area indicates the sampling time window recommended by the manufacturer.

We found an overall reduced rapid test sensitivity for *Maf 2* when compared to *Mtb* (see Fig 2 and Table 2).

**Table 2:** Percentage of samples converting to positivity over time using BD TBc ID.

	% of positive samples (95% CI)		p^#^
	*Maf 2*	*Mtb*	
	(n = 51)	(n = 122)	
**BD TBc ID**			
Day 0 (T_0_)	78.4 (65.4–87.5)	90.2 (83.6–94.3)	0.04
Day 3 (T_3_)	80.4 (67.5–89.0)	95.1 (89.7–97.7)	0.002
Day 10 (T_10_)	84.3 (72.0–91.8)	97.5 (93.0–99.2)	0.001
Day 15 (T_15_)	90.2 (79.0–95.7)	97.5 (93.0–99.2)	0.04
Day 90 (T_90_)	98.0 (89.7–99.7)	98.4 (94.2–99.5)	0.88

^#^ Two-sample test for proportions.

There was strong evidence of a difference in the time to detection between *Maf 2* and *Mtb* (P = 0.001), with *Mtb* strains having a higher rate of conversion to BD TBc ID rapid test positivity than *Maf 2* (Fig 2 and Table 2).

After controlling for age, gender, smear grade, duration spent in the MGIT automated culture system, mycobacterial growth units, and whether patients had already received some TB therapy, in a multivariable analysis, there was strong evidence of association between species and conversion to BD TBc ID positivity during the first 10 days (p<0.0001, Table 3). The rate of rapid test positivity in *Mtb*-infected patients was almost 4 times higher compared to *Maf 2*-infected patients within the first 10 days. From 10–90 days, there was no evidence of difference in the time to a positive test between *Mtb* and *Maf 2* (p = 0.15, Table 3).

**Table 3:** Multivariable analysis of BD TBc ID conversion time to positivity using interval-censored failure time regression using Weibull distribution. Each individual regression was adjusted for all other variables.

		Adjusted HR t (95% CI)	p
Testing time interval			
0–10 days	*Maf 2*	1	-
	*Mtb*	3.47 (1.75–6.88)	<0.0001
10–90 days	*Maf 2*	1	-
	*Mtb*	0.33(0.08–1.48)	0.15
Gender	Female	1	-
	Male	0.83 (0.49–1.40)	0.49
Age		0.99 (0.97–1.01)	0.13
Smear grade			0.01^a^
	Scanty	1	-
	+	1.31 (0.64–2.68)	0.47
	++	1.29 (0.63–2.63)	0.49
	+++	2.91 (1.38–6.14)	0.005
Therapy	No	1	-
	Yes	0.95 (0.62–1.46)	0.82
Time to MGIT positivity (h)		1.004 (1.002–1.004)	<0.0001
MGIT Growth units		1.00 (0.99–1.01)	0.33

^a^ Wald test assessing evidence of overall association between smear grade and time-to-conversion.

There was strong evidence of association between smear grade and conversion to positivity (p = 0.0001, Table 3) after controlling for lineage, testing time interval, gender, age, therapy, duration spent in the MGIT and mycobacterial growth units. The rate of conversion to positivity in patients having smear grade +++ was about 3 times higher than patients with a scanty smear. Generally, cultures that spent longer in the MGIT automated culture system before turning culture positive were more likely to have a BD TBc ID positive rapid test result at T_0_ (p<0.0001).

As shown in Table 3, there were no associations between conversion to BD TBc ID positivity and gender, age, therapy or mycobacterial growth units.

As only a subset of MGIT cultures were tested with both MPT64 assays, firstly, we assessed if there was any evidence of selection bias. Finding none (S2 Table), we compared their performance in a paired interval-censored analysis restricted to samples with results from both assays, correcting for the same explanatory variables as used in the BD TBc ID survival analysis.

Within each time interval and for each strain, there was no evidence of difference in time to rapid test positivity between BD TBc ID and SD Bioline (Table 4), suggesting that the decreased sensitivity may not be manufacturer specific yet intrinsic to the MPT64 target.

**Table 4:** Paired interval-censored survival analysis of conversion to positivity between BD TBc ID and SD.

	HR adjusted for testing time point (95% CI)*	p
Conversion to rapid test positivity in *Maf*		
0–10 days		
TBc	1	
SD	0.60 (0.35–1.01)	0.06
10–90 days		
TBc	1	
SD	0.86 (0.47–1.56)	0.62
Conversion to rapid test positivity in *Mtb*		
0–10 days		
TBc	1	
SD	1.39 (0.72–2.68)	0.33
10–90 days		
TBc	1	
SD	1.39 (0.72–2.68)	0.33

*: All estimates were adjusted for gender, age, therapy, smear grade, time to MGIT positivity and MGIT Growth units.

## Discussion

Our findings suggest 2.5-fold decreased expression of the *mpt64* gene in Lineage 6 of *M*. *af*ricanum compared to *M*. *tuberculosis* and a significant decrease in sensitivity (78% on day T_0_) of the MPT64 based lateral flow assays for speciation of Lineage 6 cultures relative to *M*. *tuberculosis*, as members of the MTBc.

Among the smear microscopy positive TB suspects enrolled in the present study, all were ultimately confirmed as MTBc infected, although 22% of *Maf* 2 patients, and 10% of MTB patients, would have been misclassified as NTMs if the tests had not been repeated after T_0_, the day the MGIT culture turned positive. During the 10-day MGIT positive window recommended by the BD TBc ID manufacturer, only 84% of all *Maf 2* were detected by a positive test versus 98% of *Mtb* strains. Given the relatively low cost, limited technical expertise and shorter turnaround time associated with using rapid speciation tests compared to alternative speciation methods, MPT64 rapid tests will likely remain one of the preferred options for timely diagnosis of suspected TB despite the possibility of false negative results. Therefore, a negative MPT64 result would require confirmation by an alternative method, such as molecular tests or culture on para-nitrobenzoic acid (PNB), depending on laboratory infrastructure and resources. As BD and SD Bioline MPT64 rapid tests have been on the market for over a decade now, several groups have now evaluated them. The inability of the MPT64 tests to detect strains that have lost the *mpt64* gene or acquired mutations has been reported previously [4,23]. A recent meta-analysis reported a sensitivity ≥ 95% yet studies evaluating these tests were conducted using a very limited panel of MTBc lineages, the majority belonging to *M*. *tuberculosis sensu stricto* lineages.

Two studies including *Maf* were biased by the low number of isolates evaluated compared to *Mtb* strains tested [24,25]. In their analysis of the sensitivity of the BD TBc ID test using reference strains, Yu *et al* included one *Maf* strain among 24 NTM strains, 18 mixtures of *M*. *tuberculosis* and NTM strains, 2 *M*. *bovis* strains and 1 *Nocardia spp*. strain. In the same study, 171 clinical respiratory specimen were prospectively analyzed yet these were all *M*. *tuberculosis* isolates [25]. Gaillard and co-workers, in their assessment of both the SD Bioline and BD TBc ID tests, included 20 *Maf* among 318 MTBc consisting of 242 *M*. *tuberculosis*, 53 *M*. *canettii*, 2 *M*. *bovis* and 1 *M*. *bovis* BCG Pasteur [24]. Although all *Maf* strains were detected, neither of the studies specified whether *Maf 1* or *Maf 2* isolates were tested. Interestingly, an earlier report hinted on the possibility of MPT64 rapid test sensitivity being influenced by the amount of antigen secreted by the metabolically growing MTBc cells and emphasised the need to determine factors driving secretion [26]. Notably, Gaillard and co-workers reported the production of a faint band after the standard 15 min incubation period by 8 out of 20 *Maf* strains tested during their evaluation of both the SD Bioline and BD TBc ID tests. They suggested further incubation of cultures which produce faint bands to allow production of greater amounts of antigen by the MTBc strain that could be clearly detected as positive [24]. Interestingly, in this study, we also observed an association between the duration spent by cultures in the MGIT automated culture system and positive rapid test results. The longer cultures spent in the MGIT culture system, the more likely they were to produce detectable amounts of MPT64 and therefore a positive rapid test result. Furthermore, Gagneux and colleagues detected a non-synonymous SNP in the *mpt64* gene of all isolates from *Maf 1*, which they hypothesized could affect the sensitivity of MPT64 tests in West Africa [7]. As no *Maf 1* was identified in our study, the sensitivity of the MPT64 rapid tests to detect *Maf 1* will need to be validated elsewhere. However, in a report from Nigeria, no failures of the MPT64 test were reported in detecting *Maf 1*, albeit not all positive cultures were accounted for [27].

One possible explanation for the evidence of association between high smear grade and rapid test positivity could be the presence of a greater number of actively dividing cells secreting the MPT64 antigen leading to an abundance of antigen in the growth medium. Interestingly, *Mtb* strains reportedly divide significantly faster than *Maf 2* strains [9]. Since the secretion of MPT64 has previously been linked to active cell division [1] the differential growth behaviour could also support our finding of lower detection of MPT64 rapid tests for *Maf 2*. Additionally future studies should ascertain if the observed under expression of *mpt64* in *Maf 2* was due to mutations in the gene. Whole genome sequencing will also enable us to confirm if these *Maf* 2 strains belong to a unique clone that is currently spreading in the Gambia or whether it is a general trait of *Maf 2*.

### Conclusions

Our findings indicate that MPT64 tests need to be cautiously used in settings where *Maf 2* is common. Different modifications of workflow can be considered, such as repeating the MPT64 assay after 10 days on all T_0_ MPT64 negative cultures, and molecular confirmation as MTBc vs NTMs in AFB positive cultures that test MPT64 negative. A preferred approach will be to apply the Xpert MTB/RIF assay or Line-Probe-Assays (LPA) to all AFB positive, MPT64 negative cultures. Generally, our findings strongly emphasize the need to consider strain diversity during TB product development. Our study further demonstrates that a careful evaluation and validation of novel tests before implementation, especially in regions with geographically restricted MTBC lineages, such as *M*. *africanum* in West Africa, is imperative.

## Supporting Information

S1 Table Comparison of survivor functions of the three *Mtb* lineages; East-Asian (Lineage 2), Euro-American (Lineage 4) and Indo-Oceanic (Lineage 1) for the BD TBc ID. (DOCX)

S2 Table Comparison of clinical and demographic characteristics of study participants whose samples were positive with BD TBc ID at T_0_ and were also re-tested with SD. (DOCX)

S3 Table mRNA expression and ct values for the polyketide synthesis loci and the IS6110 transposase. (DOCX)
