# Supplementary material for: Impact of the Mycobaterium africanum West Africa 2 Lineage on TB Diagnostics in West Africa: Decreased Sensitivity of Rapid Identification Tests in The Gambia
Source: PLoS Negl Trop Dis. 2016 Jul 7;10(7):e0004801. doi: 10.1371/journal.pntd.0004801 (PMC4936735; doi:10.1371/journal.pntd.0004801)
Supplement: S1 Table — (DOCX) [file pntd.0004801.s001.docx]

**S1 Table**

|  | Number of individuals | P^a^ |
| --- | --- | --- |
| East Asian | 12 | 0.55 |
| Euro American | 100 |  |
| East Asian | 12 | 0.89 |
| Indo Oceanic | 10 |  |
| Euro American | 100 | 0.63 |
| Indo Oceanic | 10 |  |

^a^ Generalized Log-rank test for interval-censored failure time.
