# Supplementary material for: Impact of the Mycobaterium africanum West Africa 2 Lineage on TB Diagnostics in West Africa: Decreased Sensitivity of Rapid Identification Tests in The Gambia
Source: PLoS Negl Trop Dis. 2016 Jul 7;10(7):e0004801. doi: 10.1371/journal.pntd.0004801 (PMC4936735; doi:10.1371/journal.pntd.0004801)
Supplement: S2 Table — (DOCX) [file pntd.0004801.s002.docx]

**S2 Table**

|  | Positive samples re-tested with SD  n = 53 | All positive samples with BD TBc ID at T_0_  n = 150 | p^#^ |
| --- | --- | --- | --- |
| Age, mean | 33.9 | 32.3 | 0.37 |
| Female, n (%) | 17 (32.1) | 43 (28.7) | 0.58 |
| *Maf*, n (%) | 18 (34.0) | 40 (26.7) | 0.23 |
| Had therapy, n (%) | 28 (52.8) | 74 (49.3) | 0.61 |

^#^: One-sample t-test for continuous variable and one-sample proportion test for binary variables. The 150 positive samples were considered as the population from which 53 positive samples were randomly selected.
