# Supplementary material for: Impact of the Mycobaterium africanum West Africa 2 Lineage on TB Diagnostics in West Africa: Decreased Sensitivity of Rapid Identification Tests in The Gambia
Source: PLoS Negl Trop Dis. 2016 Jul 7;10(7):e0004801. doi: 10.1371/journal.pntd.0004801 (PMC4936735; doi:10.1371/journal.pntd.0004801)
Supplement: S3 Table — (DOCX) [file pntd.0004801.s003.docx]

**S3 Table**

| Fold Change | | | p-value |  |  |  |  |  |  |  |  |  |  |  |
| --- | --- | --- | --- | --- | --- | --- | --- | --- | --- | --- | --- | --- | --- | --- |
|  |  |  |  | *Maf 2* | *Maf 2* | *Maf 2* | *Maf 2* | *Maf 2* | *Mtb* | *Mtb* | *Mtb* | *Mtb* | *Mtb* | *Mtb* |
| IS6110 transposase | Rv2355 | 2.60 | 0.000072 | 22.71 | 22.98 | 23.14 | 23.18 | 22.71 | 21.41 | 21.65 | 21.98 | 20.82 | 21.82 | 21.55 |
| mmpL8 | Rv3823c | 2.39 | 0.122073 | 18.98 | 20.67 | 19.65 | 19.28 | 19.69 | 18.20 | 18.57 | 20.63 | 16.93 | 19.31 | 17.79 |
| papA1 | Rv3824c | 4.53 | 0.028267 | 18.79 | 20.57 | 19.93 | 17.97 | 20.01 | 17.30 | 18.73 | 19.03 | 16.19 | 18.19 | 17.22 |
| pks2 | Rv3825c | 1.83 | 0.035326 | 19.60 | 20.10 | 18.94 | 19.26 | 20.34 | 18.12 | 18.99 | 19.02 | 18.47 | 19.16 | 16.44 |
| pks3 | Rv1180 | 16.00 | 0.000026 | 24.41 | 24.37 | 24.67 | 24.34 | 22.19 | 20.23 | 20.51 | 20.17 | 20.65 | 21.11 | 19.85 |
| pks4 | Rv1181 | 28.25 | 0.000001 | 22.99 | 23.84 | 23.23 | 23.28 | 21.70 | 18.47 | 18.48 | 18.34 | 17.59 | 19.08 | 17.37 |
| papA3 | Rv1182 | 20.68 | 0.000002 | 19.85 | 20.43 | 20.24 | 19.71 | 18.96 | 15.56 | 15.40 | 15.81 | 15.02 | 16.01 | 13.87 |
| mmpL10 | Rv1183 | 9.00 | 0.015256 | 20.28 | 21.70 | 20.09 | 20.35 | 17.49 | 17.27 | 16.30 | 16.95 | 15.76 | 17.83 | 19.81 |
